# A pilot study of an online group-based Internal Family Systems intervention for comorbid posttraumatic stress disorder and substance use

**DOI:** 10.3389/fpsyt.2025.1544435

**Published:** 2025-03-27

**Authors:** Dilara Ally, Laure Tobiasz-Veltz, Kevin Tu, Alexandra Comeau, Clare Bumpus, Tori Blot, Fiona Kate Rice, Brian Orr, Hanna Soumerai Rea, Martha Sweezy, Zev Schuman-Olivier

**Affiliations:** ^1^ Department of Psychiatry, Cambridge Health Alliance, Cambridge, MA, United States; ^2^ Department of Psychiatry, Harvard Medical School, Boston, MA, United States

**Keywords:** PTSD-SUD, addiction, trauma, Internal Family Systems, telehealth, whole-person treatment, community mental health

## Abstract

Individuals with comorbid posttraumatic stress disorder (PTSD) and substance use disorder (SUD) present with a diversity of symptoms. Current interventions show minimal efficacy differences and have high attrition. Offering a variety of treatment options, including virtual ones, ensures treatment access that is appropriate and acceptable to individual needs. We conducted a single-arm pilot study to examine the acceptability and feasibility of an online intervention based on Internal Family Systems (IFS), called the Program for Alleviating and Reducing Trauma, Stress, and Substance Use (PARTS-SUD). Ten adults (N=10), with comorbid diagnoses of PTSD and SUD, were allocated to 12 weekly groups with 6 individual counseling sessions. Our pre-specified aims were acceptability (70% overall acceptability, 75% willingness to refer a friend), and feasibility (70% completion), with key exploratory clinical outcomes (PTSD symptom severity and craving). Participants rated the intervention with a mean score of 86% on acceptability, 92% on willingness to refer a friend, retaining 70% of participants at 12 weeks. Furthermore, PTSD symptoms reduced by 1.7 points/week (95% CI: -2.45, -0.93, p=0.002) with 54% of the sample achieving a minimally important difference in PCL-5 scores. Craving Scale scores were reduced by 0.25 points/week (95% CI: -0.45, -0.06, p=0.014). An online IFS intervention was a feasible and acceptable way to provide whole-person treatment for people with PTSD-SUD within a diverse community mental health center setting. Despite being a small pilot study, decreases in both PTSD symptom severity and craving indicate the need for a randomized controlled trial with a large, diverse sample.

## 1 Introduction

Between 30-60% percent of individuals with posttraumatic stress disorder (PTSD) also have a co-occurring substance use disorder (SUD) (1). Most interventions that treat PTSD-SUD fall into one of two models: present-centered or past-focused (2). In most present-centered models (e.g., Seeking Safety (SS), Relapse Prevention (RP), and Integrated Cognitive Behavioral Therapy (ICBT)) the emphasis of the group-based modality is on substance use (except SS) with the goal of teaching coping skills (3). In contrast, past-focused models (e.g., Eye Movement Desensitization Reprocessing (EMDR), Prolonged Exposure (PE), Cognitive Processing Therapy (CPT)) are individual-based with a focus on exploring and processing traumatic memories (4). Meta-analyses of PTSD-SUD treatments find that past-focused (EMDR, PE, ICBT) treatments while superior to present-centered (RP, SS) treatments and SUD treatment-as-usual for reducing PTSD symptoms, are not necessarily more effective when examining SUD outcomes (1, 2, 5, 6). Additionally, irrespective of past or present focus, SUD outcome studies have not reported differences between group and individual modalities (7). Although trauma-focused therapies for PTSD/SUD, such as cognitive behavioral therapy integrated with prolonged exposure (COPE), are recommended treatments (in Veteran Affairs SUD programs) (8), most report high dropout rates, low engagement, and widely varied outcomes (1, 9, 10). In a randomized clinical trial comparing COPE and Relapse Prevention (RP) for military veterans (90.1% male, N=81), participants attended an average of 8 out of 12 sessions (67%) and study retention rates were 48-54%, despite substantial improvements in SUD and PTSD severity (4). Individual adjustment factors like age at trauma occurrence and trauma count may impact symptom reduction of PTSD or SUD, with one study finding that earlier trauma age predicted reduced SUD improvement while trauma count did not predict changes following either exposure-based integrated treatment of PTSD+SUD (COPE) or a SUD-focused treatment (RP) (11). Given the diversity of PTSD symptoms (e.g., intrusive thoughts, emotional dysregulation, anxiety, depression, dissociation, self-injury), it is not surprising then that different interventions may be appropriate *and acceptable* for some individuals but not others.

With the high dropout rates and low retention, there is a clear need to accommodate patient preferences, thus effective treatments of PTSD-SUD may ultimately depend on the unique preferences and values of the individual seeking care (e.g., cultural/ethnic preferences, treatment location, treatment motivation, or acceptability of the intervention) (10, 12). One qualitative study found patients preferred integrated approaches, (which integrate group and individual sessions) and interventions that “treated the whole-person” rather than focusing on addiction alone (13). Also, when participants perceived improvements in one disorder, they were more likely to experience improvements in the other, favoring simultaneous, rather than sequential treatment of SUD and PTSD (14). Seeking Safety is one of the few PTSD-SUD treatment options that offers an integrated treatment approach, considers the context of the individual, and addresses interpersonal aspects of trauma and substance use (15). Lastly, the adoption of telehealth and videoconferencing platforms for mental health interventions has increased significantly, primarily due to their ability to increase access to treatment (16). One comprehensive review focused on interventions for SUD documented both effectiveness and high patient satisfaction with telehealth-based interventions (17). A different systematic review examining technology-based interventions for those with co-occurring trauma and substance use demonstrated efficacy in reducing either trauma or substance use (16). In general, reviews indicate that interventions incorporating several key elements could substantially address public health needs. These elements include: brief duration, whole-person approach, telehealth platform delivery, concurrent treatment of PTSD and SUD through both past- and present-focused techniques, and intentional design for the engagement of diverse populations in community mental health and SUD treatment environments (10).

Internal Family Systems (IFS) is a non-pathologizing, psychotherapeutic model that merges both present- and past-focused techniques within a whole-person approach. With more than 12,000 trained clinicians in 2022, IFS is rapidly spreading and has a high level of acceptability among patients with PTSD (18, 19). IFS views the internal psyche of all individuals as an ecology of ‘no bad parts’, with each part representing a sub-personality with its own motivations, sensations, thoughts, emotions, and perceptions. These ‘parts’ are viewed as distinct from one’s core consciousness, which is characterized by non-judgement and qualities such as mindfulness, patience, compassion, and curiosity (20). Informed by insight-oriented therapeutic lineages (i.e., family systems, relational, object relations, and attachment), IFS holds that every individual carries an innate capacity for curiosity, care, mindfulness, acceptance, and love (20). In IFS the focus is not directly on the narrative specifics of the trauma, symptom management, or reducing substance use. Instead, IFS incorporates elements of present- and past-focused models through non-directed inquiry-based methods (internal narrative dialogue, contemplative practice, and visual imagery). Similar to Mindfulness-Based Relapse Prevention (MBRP) or Acceptance and Commitment Therapy (ACT), IFS cultivates present-moment awareness of thoughts, emotions and physical experiences, fostering a nonjudgmental acceptance of those experiences (21–23). Past-centered components of IFS include participant-titrated imaginal exposure to traumatic material as a way to reduce emotional sensitivity and affective dysregulation to trauma-associated environmental cues (20). Additionally, IFS views a core internal conflict among those with SUD as a polarization between disinhibition (impulsivity) and inhibition (control). This conflict is driven by “parts” as a means to manage and mitigate intense bodily sensations (e.g., throat contraction), cognitions (e.g., “I am worthless”), and affect (e.g., shame) (20). Despite a growing adoption of IFS among clinicians, there are no studies on the efficacy of IFS in groups for the treatment of comorbid PTSD-SUD nor for the treatment of SUD. Early research suggests preliminary efficacy of IFS for PTSD and related symptoms such as depression and inflammation-derived disorders (18, 19, 24, 25). A recent study demonstrated that participants diagnosed with PTSD who received a group-based intervention delivered via an online platform exhibited significant reductions in PTSD symptom severity (from Baseline to Week 16 d = -0.7, p=0.005; Baseline to Week 24 d = -0.9, p<0.001). Furthermore, the research also revealed that group-based IFS may reduce PTSD symptoms through increases in emotion regulation, self-compassion, and the ability to engage meta-awareness through decentering (19, 25).

We conducted a single-arm pilot study to examine the acceptability and feasibility of a 12-week virtual group-based program called the Program for Alleviating and Reducing Trauma, Stress and Substance Use Disorder (PARTS-SUD). PARTS-SUD, an IFS-based intervention, fills a treatment gap with its whole-person approach and telehealth platform delivery for those with PTSD and SUD. Exploratory aims were to investigate the effects of the intervention on two clinically relevant outcomes (PTSD severity and craving) using self-report surveys (PTSD Checklist for DSM-5 [PCL-5] and Substance Craving Scale) from Baseline to Week 12. According to minimally important difference (MID) metrics, a change of 9-12 points on the PCL-5 indicates real improvement in PTSD symptoms within civilian populations (26). Based on established research (26, 27), we defined clinically meaningful improvement as a 10-point reduction in PCL-5 scores by week 12, using the midpoint of the validated 9-12 range for civilian populations.

## 2 Materials and methods

### 2.1 Participants

The recruitment period spanned two weeks in September 2023. We received referrals from the Cambridge Health Alliance (CHA) network of patients, including thirty-three who were from CHA primary care providers or clinicians in psychiatry and addiction services. The remaining fifty-three referrals were CHA patients who self-referred via flyers posted online or through the CHA’s Center for Mindfulness and Compassion website. Seventy-seven total referrals were assessed for eligibility (six referrals were received after the intervention had already begun). The study team contacted all participants ≥18 years old, who were CHA patients with health insurance coverage for group psychotherapy to conduct initial eligibility screening. Thirteen declined participation and thirty-one did not respond to outreach. Patients with severe depression, psychosis, and mania were excluded. We also required that participants had a sufficient understanding of English for consent, a reliable electronic device with a sufficient data plan, and a low likelihood of hospitalization, incarceration, or giving birth within 12 weeks of the start of the online intervention.

### 2.2 Procedure

The original Program for Alleviating and Reducing Trauma and Stress (PARTS) was designed and evaluated as a hybrid (group and individual) 16-week IFS-based intervention for a PTSD population (N=15) with findings of significant reduction in PTSD symptom severity by Week 16. The original curriculum was designed for implementation in an online, hybrid integrated intervention (group and individual). Participants engaged in PARTS had 16-weeks of a 90-minute IFS-based groups with eight 50-minute individual IFS counseling sessions. While the typical length for PTSD intervention clinical trials is 16 weeks, a review of treatment literature demonstrated that treatment lengths for PTSD-SUD trials were often shorter, given the higher risk of attrition due to SUD comorbidity (2). PARTS-SUD was redesigned to consist of 12 weekly 90-minute group sessions and six 50-minute adjunct individual counseling sessions, equaling a total of 22.5 hours of treatment time. **Supplementary Table 1** outlines the 12 session topics, intervention elements, and any deviations from intervention fidelity. Both group facilitators were licensed, Level 2 trained Internal Family Systems mental health providers (LMHC and LICSW), and the LICSW was the therapist for all individual sessions.

After consent, each participant completed a baseline assessment of clinical surveys. Participants were compensated in the form of electronic gift cards up to a maximum of $140 for completion of study activities. No incentive was provided for group/individual session attendance. The first payment was made for completion of the 60-minute consent with subsequent payments when participants filled out each set of clinical surveys at baseline, weeks 4, 8 and 12. Time incentives were also provided to increase response rates ($10 for survey completion within 24hrs, $5 if within 48hrs). All participants completed and signed informed consent forms. REDCap software captured informed consent forms and all survey data. This study was approved by CHA’s Institutional Review Board (IRB) (CHA-IRB-23-24-237) and registered at ClinicalTrials.gov (NCT06207409). Adverse event reports were collected at various points throughout the study using a clinical survey asking about adverse health outcomes encountered during the past four weeks as well as engagement calls by study staff. Adverse event (AE) reports collected weekly and post-intervention were reviewed by the Principal Investigator and reported to the CHA IRB and an independent monitor.

### 2.3 Measures

*Screening:* This study utilized several validated instruments to assess key variables. To screen for study SUD eligibility, we used two scales to assess drug and alcohol usage. The first was the Brief Addiction Monitor (BAM)- Use Frequency subscale, which is a 4-item instrument with good internal consistency, previously validated in diverse clinical populations (28). We used the PhenX Toolkit subscales: Substances – Lifetime; Age of First Use; Drugs and Alcohol – 30-Day quantity/frequency. We also used the Computerized Adaptive Test for Mental Health to screen for depression, anxiety, PTSD and substance use risk (29). Baseline demographics (**Table 1**) were obtained from electronic health records.

**Table 1:** Screening and baseline characteristics of those enrolled in the study.

Screening and Baseline Characteristics	N=10
PCL-5, Mean ± SD	47.5 ± 15.5
Individual Trauma Load, LEC-5, Mean ± SD	7.0 ± 2.1
ITQ-DSO, Mean ± SD	14.8 ± 6.0
CAT-MH	
Depression, Mean ± SD	71.5 ± 11.6
Anxiety, Mean ± SD	70.9 ± 18.2
Substance Use Disorder, Mean ± SD	62.0 ± 12.3
PTSD, Mean ± SD	60.5 ± 13.2
Brief Addiction Monitor, Usage Mean ± SD	6.4 ± 6.8
Lifetime Usage of Different Drugs	
Sedatives or tranquilizers	7.0 (70.0%)
Opioid painkillers	6.0 (60.0%)
Marijuana	9.0 (90.0%)
Cocaine or crack	4.0 (40.0%)
Stimulants	4.0 (40.0%)
Club drugs	4.0 (40.0%)
Hallucinogens	3.0 (30.0%)
Heroin/Fentanyl	1.0 (10.0%)
Age of First Drug Use, Mean ± SD	18.7 ± 4.1
Days of drug use in the past 30 days, Mean ± SD	17.5 ± 13.1
Age ± SD	44.5 ± 16.8
Gender, N (%)	
Male	4 (40%)
Female	4 (40%)
Non-binary	2 (20%)
Race and Ethnicity, N (%)	
White	4 (40%)
Hispanic/Latinx/a/o	4 (40%)
Asian	2 (20%)
Employment Status	
Full-time employment	3 (30%)
Part-time employment	3 (30%)
Retired	2 (20%)
Unemployed	2 (20%)

To be eligible for participation in the study, individuals were required to have a diagnosis of PTSD *or* Complex PTSD *and* be experiencing a substance use disorder. This eligibility criterion led to the exclusion of one participant who, despite having a PTSD diagnosis, did not meet criteria for SUD. This resulted in a total of N=10 participants.

*Acceptability and Feasibility*: To assess the acceptability of the intervention, we used three different surveys: a 10-item questionnaire utilizing the Theoretical Framework of Acceptability (TFA) completed at week 12; a 12-item Satisfaction Survey, including the willingness to refer a friend item, which was completed at week 12; a 4-item Confidence and Credibility of Intervention completed at baseline, weeks 4, 8 and 12 (30). The study defined acceptability as a mean score of >3.5 (Total=5) on the TFA questionnaire, general acceptability item; and a mean score >3.75 (Total=5) on willingness to refer a friend. Feasibility was defined as 70% of participants completing at least 75% (9/12) of groups (intervention feasibility), and 70% of participants completing post study assessments (9/12) (study protocol feasibility). At week 12, we collected free text comments in the Satisfaction Survey. Participants provided feedback about the following: least favorite/favorite aspects of the program, most important thing learned, hardest aspect of the program, and suggestions for change.

*Exploratory Quantitative Outcomes:* At baseline, weeks 4, 8 and 12, we also collected exploratory outcomes including clinical outcomes of symptoms of PTSD, complex trauma, and craving as well as additional clinical outcomes, including depression, anxiety, and dissociation symptoms. PTSD severity was assessed using the 20-item PCL-5 with scores ranging from 0-80 (31). The PCL-5 shows strong internal consistency (α=0.94), convergent validity with other PTSD measures (r=0.84-0.85) (32). Complex PTSD symptoms were assessed using 6-items from the International Trauma Questionnaire - Disturbances of Self-Organization (ITQ-DSO), ranging from 0-24 (33). We used the Craving Scale, a brief, 3-item self-report measure of craving which had items that ranged from 0-10 for a total possible score of 30 (34). This scale was previously validated with those seeking treatment for SUD, demonstrating strong internal consistency (α=0.81), reliability for both alcohol and opioid use disorder (α=0.78 and 0.80), a single factor latent structure, as well as strong concurrent and discriminant validity (34). Additionally, the Craving Scale was strongly associated with specific BAM items which assessed craving intensity and perceived self-efficacy in maintaining abstinence (23, 34). Using PROMIS measures, we assessed anxiety with a 7-item scale and depression with 8-item version, with both scored using a standardized T-score (35). The Multiscale Dissociation Inventory is a 30-item scale measuring frequency of dissociative symptoms (36).

Mechanisms explored included emotion regulation, decentering, and nondual awareness. Emotion regulation was assessed using the Difficulties in Emotion Regulation (DERS), a 36-item scale ranging from 36-180 (37). Decentering was measured using the 11-item Decentering subscale from the Experiences Questionnaire, ranging from 11-55 (EQ-D) (38). Nondual awareness was evaluated with a 13-item Nondual Awareness Dimensional Assessment scale (NADA), ranging from 13-65 (39).

### 2.4 Data analysis

Baseline outcomes, demographics, primary acceptability and feasibility outcome measures were summarized using univariate descriptive statistics. To understand the potential impact of Treatment Time (baseline to Week 12) on primary/secondary outcomes, we used two different linear mixed-effects model (LMEM) analyses. In the first, Treatment Time was treated as a continuous fixed effect (global effect), allowing for the estimation of a global trend across participants for exploratory clinical outcomes (PCL-5 and Craving Scores). This analysis included both types of random effects (intercept and slope) in order to capture individual variation inherent in how participants may respond to the intervention (initial state and rate of change) (40). In the second type of LMEM analysis, Treatment Time was treated as a factor with only random intercepts to enable comparison with existing literature. Results from both models are reported, however, models with maximal random-effect structures (intercept and slopes) are preferred because failure to include both can inflate Type I error rates by underestimating the standard errors of the fixed effects (40). To quantify uncertainty around the marginal effect of the Treatment Time, we employed a non-parametric bootstrapping procedure (N=1000). For each bootstrap iteration, we resampled participants (N=10) with replacement from the baseline pool, effectively simulating datasets with maximal attrition. In cases where the linear mixed-effects model failed to converge we explored alternative model specifications, e.g., assuming no correlation among random slope and intercept terms by setting the covariance parameter to zero (40).

## 3 Results

### 3.1 Participant flow and baseline characteristics

**Figure 1:**
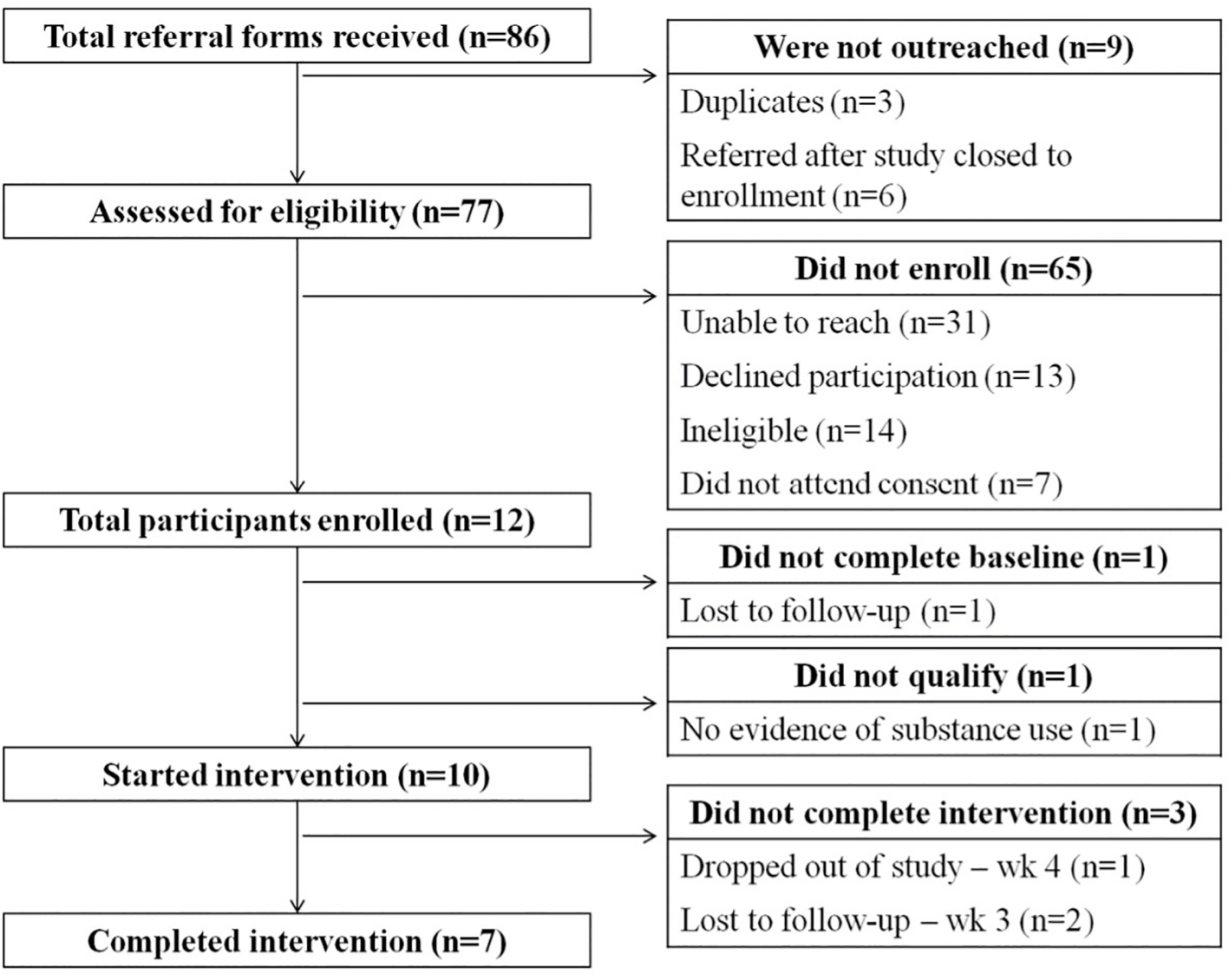
CONSORT diagram.

Over two weeks of recruitment, providers referred 86 patients, seventy-seven were assessed for eligibility. Nineteen consent sessions were scheduled, and twelve enrolled in the study and provided informed consent (**Figure 1**). One participant was lost to follow-up after consent before baseline assessments and one was consented but subsequently excluded because of insufficient evidence of SUD. Ten participants (N=10) were allocated to the study, completed the screening and baseline measures, and were enrolled in the 12-week intervention. Descriptions of baseline and demographic characteristics can be found in **Table 1**. Notable characteristics of our sample included an average of 7.0 lifetime traumatic events (SD=2.1) on the LEC-5 (**Supplementary Table 2**), with 90% of participants experiencing sexual/physical assault. Mean 30-day alcohol usage was 8 days (SD=9.0), with a mean 2.3 drinks per day (SD=2.7), while average 30-day drug use was 17.5 days (SD=13.1) with marijuana, opioid painkillers and sedatives as drugs most frequently used (**Table 1**).

### 3.2 Acceptability, feasibility, and attrition bias

The study aims focused on two aspects of acceptability: affective attitude and satisfaction. At week 12, we found a mean rating of 4.3/5 (86%, SD=0.5) by completers (N=7, 70%) on the TFA general acceptability question about the PARTS-SUD program. Among those participants who completed a satisfaction survey at Week 12 (N=7), the mean score was 4.6/5 (92%, SD=0.5) on willingness to refer a friend. As participants advanced through the program, they became more likely to recommend it to a friend (**Table 2**.) Overall ratings of the facilitator(s) and group were also high at 98% (4.9/5, SD=0.4) and 88% (4.4/5, SD=0.5), respectively. See **Supplementary Tables 4–6** for detailed mean confidence, credibility, satisfaction, and acceptability scores. In terms of study feasibility, of 10 participants enrolled in the study, seven participants (70%) completed the post-intervention assessments (**Table 2**). One participant formally withdrew from the study at week 4 and two were lost to follow-up. Seven participants attended at least 8 group sessions and six participants attended 9 of 12 group sessions. Seven participants attended 100% of the 6 individual sessions. Each weekly group had a mean attendance of 6 participants (SD=1.0). Participants who did and did not withdraw from the study did not differ in demographic characteristics. Univariate statistics for non-completers are presented in **Supplementary Table 7** for all clinical outcomes at baseline.

**Table 2:** Acceptability and feasibility outcomes.

Type	Question	W0	W4	W8	W12
Acceptability					
Affective attitude	How acceptable was the PARTS intervention to you? (Scale: 1-5)	–	–	–	4.3 (0.5)
Satisfaction	How confident would you be in recommending this program to a friend who experiences similar problems? (Scale: 0-8)	5.2 (1.8)	5.5 (1.8)	6.9 (1.4)	7.0 (1.0)
	I would recommend this program to a friend. (Scale: 1-5)	–	–	–	4.6 (0.5)
Intervention credibility	At this point, how successful do you think this program will be in reducing your distress symptoms? (Scale: 0-8)	5.8 (1.4)	5.2 (1.8)	6.4 (1.2)	–
Perception of burden	Engaging with the PARTS intervention interfered with my other priorities. (Scale: 1 to 5)	–	–	–	2.3 (0.5)
Feasibility					
Intervention	Number of Participants Retained	10	10	8	7
Study Protocol	Survey Completion Rate	10	10	8	7

Acceptability was measured in four different ways (affective attitude, intervention credibility, intervention satisfaction, and perception of burden). Study aims focused on two of these: affective attitude and satisfaction. The feasibility of the intervention and study protocol were assessed.

### 3.3 Exploratory quantitative measures

**Figure 2:**
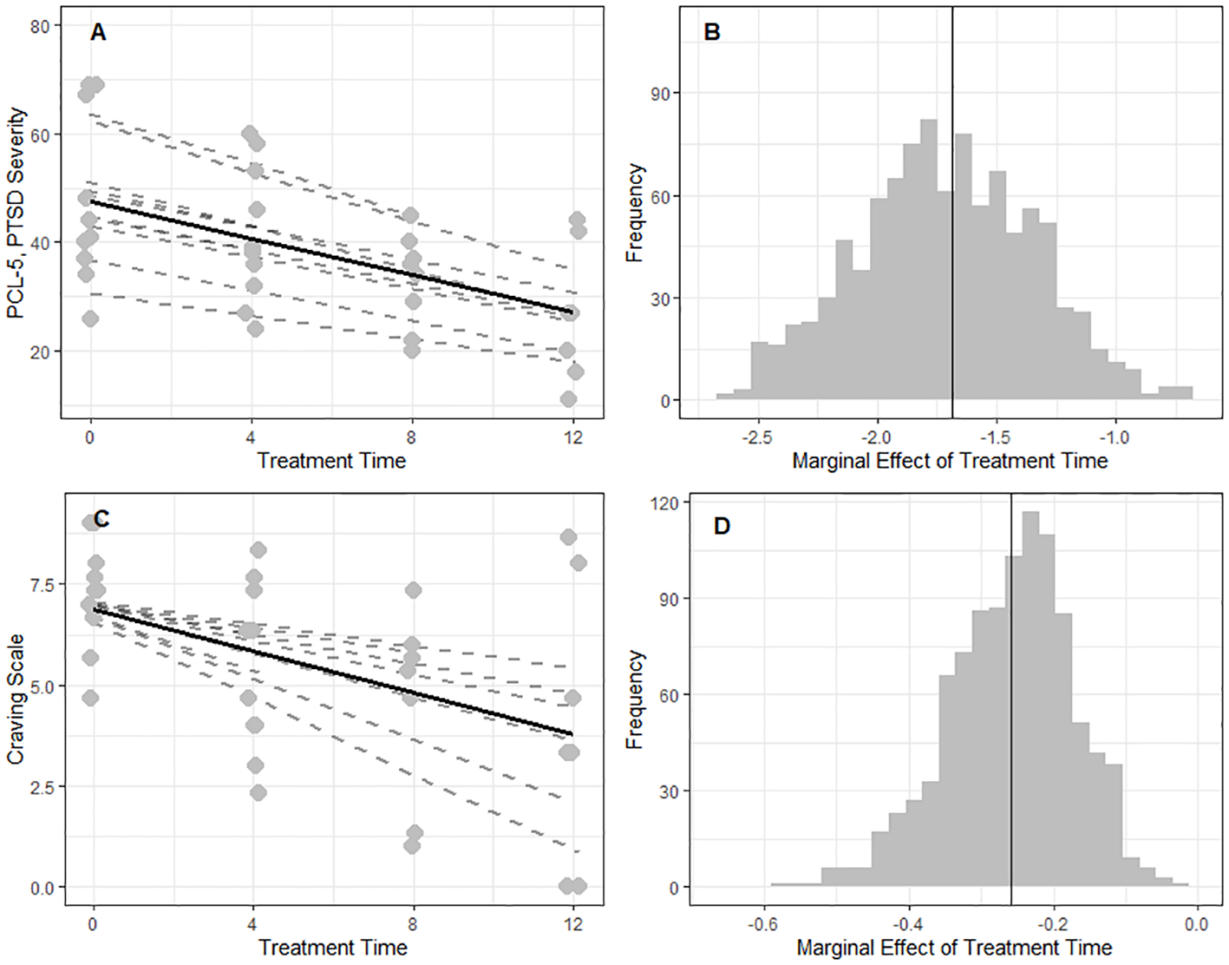
Global effects for the reduction in symptom severity of PTSD and craving were observed over the course of the intervention. A linear mixed-effects analysis was conducted to examine the effect of intervention time on two clinical outcomes of interest (PCL-5 and Craving Scale). Treatment Time was treated as a continuous fixed effect (marginal), allowing for the estimation of a global trend (solid black line) across participants. To account for individual differences among participants (N=10), both at baseline and in the rate of change over time random intercepts and random slopes (dotted grey lines in **(A, C)** were included in the model. To understand the uncertainty around the marginal effect of intervention time (*β*, solid black line), a non-parametric bootstrapping procedure (N=1000) with replacement (including attrition) was performed **(B, D)**. **(A)** PCL-5: There is a global effect of Treatment Time (*β* = -1.7 ± 0.4, *p* = 0.002). **(B)** PCL-5: In 52% of the shuffled datasets we found a marginal effect at least as extreme as in the observed sample. **(C)** Craving Scale: A global effect of Treatment Time (*β* = -0.3 ± 0.1, *p=0.014*) with higher participant variability in rates of change over time. **(D)** Craving Scale: In 30% of the shuffled datasets we found a marginal effect at least as extreme as in the observed sample.

Fifty-four percent (out of baseline total N=10) of our sample achieved a minimally important difference (MID) in PCL-5 scores (26). From a linear mixed-effect model (LMEM) with Treatment Time as a continuous variable and the inclusion of maximal random effects, we found PCL-5 scores (PTSD symptom severity) declined by 1.7 (SE=0.4) points/week (95% CI: -2.45, -0.93, *p*=0.002) (**Figure 2A**). A global decrease of 0.25 points/week (SE=0.09) was also observed for craving, as a function of Treatment Time (95% CI: -0.45, -0.06, p-value=0.014) with higher participant variability in rates of craving change, as evidenced by the variable random slopes (**Figure 2C**). We also observed significant declines of 0.7 points/week (SE=0.3) in the International Trauma Questionnaire’s Disturbances of Self Organization (ITQ-DSO) (95% CI:-1.3, -0.04), 0.53 points/week (SE=0.14) in PROMIS-Anxiety (95% CI: -0.8. -0.2), and 0.71 points/week (SE=0.14) in PROMIS-Depression (95% CI: -0.99, -0.4) (**Supplementary Table 3**). After correction for multiple tests, outcomes from a random intercept LMEM were comparable to a maximal random effect model (random slopes and intercepts) (**Supplementary Table 3**).

## 4 Discussion

This is the first study to test the acceptability and feasibility of an online Internal Family Systems intervention for a population with comorbid PTSD-SUD. These findings show comparable levels of acceptability and feasibility to other evidence-based PTSD-SUD interventions (1). A recent systematic review found in past-focused treatments 62% of participants with PTSD-SUD were considered completers compared to only 51% in present-centered ones (1). Another meta-analysis reported that treatment session completion rates were 52.11% (SD=20.86) in past-focused PTSD-SUD interventions and 50.73% (SD=10.27) for present-focused ones (5). In this study, 70% completed post-intervention assessments and completed at least 8 groups and 6 individual sessions.

Consistent with findings in the literature, our study participants reported that the most beneficial aspect of the program was the combination of group and individual support (13, 14). Reasons cited included the ability to engage in an active and personal reflection process and participate in an interactive experiential group. Finally, while it was clearly apparent to participants how the PARTS-SUD intervention would help alleviate symptoms of PTSD, they were only mildly clear on how it would address substance use (**Supplementary Table 6**). This offers an opportunity for intervention optimization, clarifying for participants how IFS can support the reduction of addiction-related behaviors, thoughts, and emotions. As with both past and present-focused PTSD-SUD interventions, our study demonstrated observable changes in PTSD symptom severity (1, 3, 5).

Despite individual differences in the rate of change in substance craving, one remarkable result was the global decline in substance craving levels. Craving is considered a core component of addiction, with a diverse and large body of research highlighting the role of stress exposure triggering craving among individuals (23, 41). Some studies suggest that intrusive memories or traumatic cues activate increased craving causing relapse, while others have shown craving is elicited by a negative emotional state and substance use constitutes a coping strategy (41). From an Internal Family System perspective, there are two primary dialectical tensions that characterize the intrapsychic system. The initial tension manifests between exiled self-states, characterized by intrusive memories or intense negative emotional states, and the protective subsystems that emerge in response to these vulnerable elements (20). The secondary tension exists within the protective configuration itself, specifically between inhibitory regulatory mechanisms (‘managers’), which maintain a homeostasis through ruminative thoughts and/or behavioral constraint, and dysregulatory mechanisms (‘firefighters’), which seek affective modulation through behavioral disinhibition and/or substance use. The reduction in reactivity to the intense emotional states may be achieved by a two-stage constraint release process in IFS. An initial “unblending,” stage generates meta-awareness, self-trust, inner compassion for *all* parts, including impulsive parts, recovery manager parts, and self-critical parts that emerge after abstinence violations. In the second phase, “unburdening,” a patient-controlled imaginal trauma exposure and rescripting reduces emotional sensitivity to trauma-based environmental triggers (20). One suggestion is that increased awareness of sensations, thoughts, and emotions combined with reduced avoidance led to nonreactivity over time that may explain observed reductions in craving (41).

Although this study demonstrated acceptability among a small sample of patients with PTSD-SUD, there were several notable design and implementation limitations. First, our sample size (N=10) likely did not adequately represent the full distribution of substance use, potentially leading to an overestimation of effect sizes and lack of generalizability. Secondly, since we did not have a control group in this single-arm study, we cannot know definitively if the symptom reduction was a result of time, non-specific intervention effects, or regression to the mean. Third, although we tested the assumption of linearity in our data, it is unlikely that all individuals follow the same gradual linear decline in PTSD symptoms or craving (42). Fourth, for this acceptability and feasibility study, we only collected longitudinal data about substance craving, and did not collect validated objective measures of substance use frequency (e.g., regular toxicology testing, 30-day timeline follow back for substance use). Fifth, we only sampled four timepoints over the 12-week intervention; future studies should utilize additional timepoints with toxicology testing and substance use reporting during the intervention and with follow-up timepoints to understand whether early linear declines are sustainable over longer periods of time and if the intervention is a continued driver of symptom improvement and substance use reduction. Although our participant characteristics were like past PTSD-SUD studies and included 60% minoritized subjects (5) no African American/Black participants were allocated after baseline screening for SUD. Lastly, our telehealth program may limit access to clinical populations who experience financial and/or housing instability and cannot afford smartphones, tablets or computers, in which case in-person PARTS groups could be appropriate. Due to these limitations, this study represents a first step toward implementing a larger randomized controlled trial of an IFS-based hybrid program.

In conclusion, integrated treatment modalities that consider real-world implementation factors like diagnostic complexity, a patient’s priorities, values, burdens, and preferences, as well as treatment access, efficacy and cost-effectiveness, would be widely welcomed in public community mental health clinics for those with PTSD-SUD (10). This work with the IFS model stems from recognition of a need for an even more trauma-sensitive approach that can engage similar mechanisms of behavior change as mindfulness and compassion-based interventions through ways that are easier for patients with PTSD to access (43). Paradoxically, with its emphasis on ‘parts,’ the IFS paradigm and PARTS-SUD “treat the whole person,” not just the trauma or addiction (13). Identifying and understanding parts and their conflicts may enable decentering, self-regulation, and states of non-judgmental acceptance (44). In offering a combination of virtual individual and group sessions to simultaneously treat PTSD/complex PTSD-SUD, the PARTS-SUD intervention is an integrated treatment modality which considers patient preferences, values, and social context. Importantly, IFS and PARTS-SUD enabled at least one participant to reflect: “[I] face my dark parts with curiosity and compassion. I learned that my true self is good and wise, that the answers will reveal themselves if I am patient, persistent and present help is on the way! I learned to listen to and trust myself.”

## Data Availability

The original contributions presented in the study are included in the article/**Supplementary Material**. Further inquiries can be directed to the corresponding author.
