# Supplementary material for: A pilot study of an online group-based Internal Family Systems intervention for comorbid posttraumatic stress disorder and substance use
Source: Front Psychiatry. 2025 Mar 27;16:1544435. doi: 10.3389/fpsyt.2025.1544435 (PMC11983591; doi:10.3389/fpsyt.2025.1544435)
Supplement: Supplementary file 1 [file DataSheet1.pdf]

Supplemental Table 1. Weekly group session themes. All group-based sessions were video recorded and rated for fidelity (high, medium, low adherence) to the curriculum by an independent clinician. Curriculum deviations are noted with an asterisk (*). Due to low group attendance during a holiday, the topic of polarization was revisited in the following session.

Week	PARTS-SUD Content Focus	Therapeutic Elements
1	Basic Concepts of IFS	Building group cohesion
2	Overview of IFS and Trauma/Substance Use	Basic concepts of IFS<br>Unblending experiential
3	Finding Self – Curious, Clear, Compassionate	Guided meditation for unblending<br>Exercise on qualities of Self
4	Unblending: Parts Separate from Self (Part 1)	Trailheads Exercises<br>Guided meditation for unblending
5	Understanding and Working with Polarizations*	Relationship between trauma and substance use<br>Exercises on Polarizations
6	Getting Permission from Parts*	Centering Exercise
7	Unblending: Parts Separate from Self (Part 2)	Exercises on speaking for not from parts
8	Legacy Burdens and Gifts	Tracking and sharing legacy burdens
9	Deepening Self Energy (Part 1)	Be with Your Heart Exercise
10	Deepening Self Energy (Part 2)	Centering Exercise
11	Speaking for Parts and Listening from Self	Centering Exercise
12	Loving your parts as the Journey Continues	Reflections on group experience

Supplemental Table 2. Screening outcomes for the Life Events Calendar 5 (LEC-5).

**LEC-5 Experienced Events, Total (N = 10)**	***N (%)***
Natural disasters (flood, tornado, earthquake, etc.)	3 (30%)
Fire or explosion	3 (30%)
Transportation accident (car accident, boat accident, train wreck, etc.)	6 (60%)
Serious accident at work, home, or during recreational activity	3 (30%)
Exposure to toxic substance (dangerous chemical, radiation)	2 (20%)
Physical assault (being attacked, hit, slapped, etc.)	9 (90%)
Assault with a weapon (being shot, stabbed threatened with a weapon)	5 (50%)
Sexual assault	9 (90%)
Other unwanted or uncomfortable sexual experience	9 (90%)
Combat or exposure to a warzone (in military or as a civilian)	1 (10%)
Captivity (being kidnapped, abducted, held hostage, prisoner of war)	2 (20%)
Life threatening illness or injury	4 (40%)
Severe human suffering	2 (20%)
Sudden violent death (homicide, suicide)	2 (20%)
Sudden accidental death	0 (0%)
Serious injury, harm, or death you caused to someone else	2 (20%)
Any other very stressful event or experience	8 (80%)

Supplemental Table 3. Exploratory outcomes and potential secondary mechanisms. Results from two different linear mixed effect models. On the left-hand side of the table, Treatment Time is viewed as a continuous variable and random slopes and intercepts were included in the model. On the right-hand side, effect sizes and estimates from the random intercept model where Treatment Time was a factor is presented for benchmarking purposes.

	Intercept		Estimate Treatment Time		Estimate Difference of Week 12 - Baseline			
Outcome	$Y_{si} = (\beta_0 + S_{0s}) + (\beta_1 + S_{1s})X_i + \varepsilon_i$				$Y_{sij} = (\beta_0 + S_{0s}) + \beta_{1j}X_i + \varepsilon_i$			
	β_0	(SE)	β_1	(SE)	$\beta_{1,W12}$	(SE)	g	[95% CI]
PCL-5	47.5	(4.2)	-1.7	(0.4)**	-18.4	(4.5)***	-1.4	[-2.7, -0.6]
Craving Scale†	6.9	(0.5)	-0.3	(0.1)*	-3.2	(0.7)**	-1.2	[-3.6, -0.1]
ITQ-DSO	54.1	(1.9)	-0.7	(0.3)*	-8.5	(2.9)***	-1.1	[-2.6, -0.2]
MDI	65.5	(3.4)	-0.7	(0.5)	-10.0	(4.7)*	-0.7	[-4.1, 0.2]
PROMIS Anxiety	26.8	(1.5)	-0.5	(0.1)**	-6.6	(1.5)***	-1.4	[-2.8, -0.6]
PROMIS Depression	29.5	(0.9)	-0.7	(0.1)**	-8.5	(1.6)***	-1.6	[-3.2, -0.7]
DERS	102.6	(4.9)	-0.8	(0.5)	-5.0	(4.4)	-0.1	[-1.6, 0.8]
EQ-D†	30.9	(1.8)	0.5	(0.3)	5.9	(2.1)*	1.1	[0.3, 2.4]
NADA†	27.3	(2.0)	0.2	(0.5)	3.6	(2.1)	0.4	[-0.7, 1.7]

NOTE: p<0.05*, p<0.005**, p<0.001***; after a Bonferonni correction, two outcomes previously signficant at an $\alpha = 0.05$, MDI and EQ-D are no longer signficant. This corresponds to the significance found in the model with both random slope and intercept. †To achieve convergence of linear mixed-effects models when singularity is present, we assumed no correlation between random effects and conducted a Bayesian analysis.[31] Coefficients of the model were not impacted by assuming $\rho = 0$ and were similar to those obtained from a Bayesian analysis (see text for details). PTSD Scale for DSM-5 (PCL-5), International Trauma Questionnaire-Disturbances of Self-Organization (ITQ-DSO); Multidimensional Dissociation Inventory (MDI); Difficulties in Emotion Regulation Scale (DERS); Experiences Questionnaire-Decentering (EQ-D); Non-Dual Awareness Dimensional Assessment (NADA).

Supplemental Table 4. Mean Confidence and Credibility of intervention.

Question	Mean Rating (SD)			
	W0	W4	W8	W12
1. At this point, how logical does the program offered to you seem?	6.2 (1.2)	5.9 (1.7)	6.5 (1.4)	NA
2. At this point, how successful do you think this program will be in reducing your distress symptoms?	5.8 (1.4)	5.2 (1.8)	6.4 (1.2)	NA
3. How confident would you be in recommending this program to a friend who experiences similar problems?	5.2 (1.8)	5.5 (1.8)	6.9 (1.4)	7.0 (1.0)
4. By the end of the program, how much improvement in your symptoms do you think will occur?	55.5 (22.5)	52.0 (19.8)	69.0 (16.4)	NA

* Question 1 and 2 rated on a scale from 0 (“Not Logical”) to 8 (“Very Logical”), Question 3 rated on a scale from 0 (“Not Confident At All”) to 8 (“Very Confident”), and Question 4 rated from 0-100%.

Supplemental Table 5. Satisfaction Scale (N=7). Rating for the Satisfaction scale was on a 5-point Likert scale from 1 (“Strongly Disagree”) to 5 (“Strongly Agree”). Open text comments indicated that the most positive aspects of the intervention included how it helped “to feel less alone about my situation,” “hearing everyone’s point of view”, the “vocabulary helped put words to my feelings and parts”, “being able to access parts of myself I’d forgotten or didn’t even know were there”, and “once you have the tools to look within and process your emotions, things get a bit easier.“ Challenging aspects of the program included “asking some parts to step back,” the centering exercise, length of surveys, technical challenges related to online video conference, “learning to listen to the pain of others with curiosity and compassion,” and scheduling conflicts during the workday.

Question	Mean Rating (SD)
	Week 12
1. I found this program helpful.	5.0 (0.0)
2. The group was well organized.	5.0 (0.0)
3. The group leader cared about me as a person.	5.0 (0.0)
4. I was able to participate and express myself in the group.	5.0 (0.0)
5. The group leader(s) were authentic, honest and real.	5.0 (0.0)
6. I learned what I was hoping to learn.	4.1 (0.7)
7. The group leader(s) had good timing when providing examples.	4.9 (0.4)
8. I would be willing to participate in the program again were it possible to do so.	4.6 (0.5)
9. The group leader was easy to understand.	4.9 (0.4)
10. I would recommend this program to a friend.	4.6 (0.5)
11. Overall Leader Rating	4.9 (0.4)
12. Overall Group Rating	4.4 (0.5)

Supplemental Table 6. Mean scores of Acceptability using the Theoretical Framework of Acceptability. (N=7)

Question	Mean Rating (SD)
	Week 12
1. How comfortable did you feel engaging with the PARTS intervention?	4.3 (0.5)
2. How much effort did it take to engage with the PARTS intervention?	3.0 (1.3)
3. How fair is the PARTS intervention for people with substance use and PTSD?	4.1 (0.7)
4. The PARTS intervention has improved my symptoms of substance use.	3.6 (0.5)
5. The PARTS intervention has improved my symptoms of PTSD.	3.7 (1.0)
6. It is clear to me how PARTS will help improve my substance use.	3.4 (0.8)
7. It is clear to me how PARTS will help improve my PTSD.	4.1 (0.4)
8. How confident did you feel about engaging with the PARTS intervention?	3.4 (1.0)
9. Engaging with the PARTS intervention interfered with my other priorities.	2.3 (0.5)
10. How acceptable was the PARTS intervention to you?	4.3 (0.5)

Likert scale for Q1: 1=Very uncomfortable, 5= Very comfortable; Q2: 1=No effort and 5= Huge effort; Q3: 1=Very unfair and 5=Very Fair; Q4-Q7 and Q9: 1=Strongly disagree and 5= Strongly agree; Q8: 1=Very unconfident and 5 =Very confident; Q10: 1=Completely unacceptable and 5= Completely acceptable

Supplemental Table 7. Attrition Bias. We provide univariate statistics of baseline measures for non-completers compared to completers without performing a Fisher's Exact test, since it has a threshold sample size of five.

Outcome	Completers Mean (SE) N=7	Non-Completers Mean (SE) N=3
PCL-5	44.9 (9.6)	53.7 (7.8)
ITQ-DSO	25.0 (2.7)	21.3 (3.8)
MDI	61.6 (3.8)	68.3 (5.2)
BAM – Usage	3.9 (1.7)	2.3 (1.5)
Craving Scale	7.4 (1.0)	6.9 (0.7)
PROMIS - Anxiety	26.0 (2.0)	29.7 (1.9)
PROMIS - Depression	29.6 (1.7)	28.3 (1.3)
DERS	103.3 (4.6)	91.7 (11.0)
EQ-D	34.0 (2.4)	22.7 (0.9)
NADA	31.0 (4.3)	22.7 (4.3)

Abbreviations for Scales:PTSD Scale for DSM-5 (PCL-5), International Trauma Questionnaire-Disturbances of Self-Organization (ITQ-DSO); Multiscale Dissociation Inventory (MDI); Brief Addiction Monitor – Usage (BAM-Usage); Difficulties in Emotion Regulation Scale (DERS);Experiences Questionnaire-Decentering (EQ-D); Non-Dual Awareness Dimensional Assessment (NADA).

Supplemental Table 8. Adverse Event Information, Relatedness & Expectedness.
We follow Office for Human Research Protection's 2007 guidance for defining and assessing adverse events and serious adverse events. Details are provided for the types of adverse events, in order of frequency at which each type was reported, including information on relatedness to the intervention and/or study procedures and expectedness. Expectedness was determined based on the participants' previous medical history and the potential risks listed in the study's Informed Consent form.

Adverse Event Type	Relatedness	Expectedness	Event Count
Physical illness, injury, or worsening of baseline physical symptoms	Unlikely or not related to the intervention or study procedures	Expected or unexpected	2
Worsening baseline psychiatric symptoms	Possibly or probably related to the intervention	Expected	1
Worsening baseline psychiatric symptoms	Unrelated to the intervention or study procedures	Not Expected	0
Worsening baseline psychiatric symptoms	Probably related to study procedures (e.g. symptoms during clinical surveys)	Expected	1
